# Smell Decline as a good Predictor of Sinonasal Polyposis Recurrence after Endoscopic Surgery

**Published:** 2016-03

**Authors:** Mahdi Bakhshaee, Mohammad Reza Sharifian, Amir Hossain Ghazizadeh, Kianoosh Nahid, Karim Jalaeian Samani

**Affiliations:** 1 *Sinus and Surgical Endoscopic Research Center, Ghaem Hospital, Faculty of Medicine, Mashhad University of Medical Sciences, Mashhad, Iran.*; 2 *Department of Otorhinolaryngology Head and Neck Surgery, Faculty of Medicine, Shahid Beheshti University of Medical Sciences, Tehran, Iran.*; 3 *Department of Otorhinolaryngology Head and Neck Surgery, Ghaem Hospital, Mashhad University of Medical Sciences, Mashhad, Iran.*

**Keywords:** Asthma, Allergic rhinitis, Endoscopic sinus surgery, Nasal secretion, Nasal obstruction, Recurrence, Sinonasal polyposis, Smell.

## Abstract

**Introduction:**

To evaluate the most sensitive symptom to predict early recurrence of nasal polyposis. Prospective longitudinal cohort study. Tertiary university referral center with accredited otorhinolaryngology residency programs.

**Materials and Methods:**

In this prospective study, we evaluated 62 patients with diffuse nasal polyposis. All patients underwent functional endoscopic sinus surgery. The author-devised questionnaire relating to the four major symptoms of chronic rhinosinusitis were answered by patients at the pre-operative visit and at 1, 3, 6, 12, and 24 months after surgery. Patients were followed up with serial endoscopic examinations, and a computed tomography (CT) scan was performed if indicated.

**Results:**

All 62 patients (37 male, 25 female) completed the study. The mean age was 41.24 ± 12.47 years. All major symptoms showed significant improvement after surgery (P=0.000); however, the severity of symptoms gradually increased in patients with a recurrence of polyposis, but at different points in time (P= 0.008). Sense of smell was the first symptom to deteriorate in patients with relapse (mean, 6 months) followed by nasal secretion (12 months), obstruction and pain(24 months). Patients with asthma, Samter’s triad, allergic fungal rhinosinusitis (AFRS) and allergic rhinitis showed symptoms of recurrence sooner than other patients (P<0.05).

**Conclusion:**

The most sensitive symptom for the early detection of recurrence of nasal polyposis is a decrease in the sense of smell. Nasal obstruction and facial pain were observed in the late stage of relapse when frank polyposis formation was established.

## Introduction

Sinonasal polyposis (SNP) is a chronic inflammatory disorder of the nasal cavity and paranasal sinus mucosal membranes that typically affects patients bilaterally in the form of a benign edematous mass known as a polyps, extending from the paranasal sinuses towards the nasal cavity (1). The etiology of SNP remains uncertain and the precise prevalence is not well known; however prevalence has been reported through medical records as varying between 0.2–5.6% (2-7).

An increase in prevalence of SNP with patient age and male gender has been observed (8). Factors or associated conditions include smoking, allergy, asthma, fungal sensitivity, nonsteroidal anti-inflammatory drug (NSAID) intolerance and genetic factors (8,9), although the relationship between SNP and allergy and the genetic-hereditary factors has yet to be clarified (8). No association between SNP and smoking has yet been demonstrated. 

Diagnosis of SNP is based on the presence of major symptoms (nasal obstruction, altered smell, anterior and/or posterior rhinorrhea, and pain or facial pressure) and minor symptoms (sore throat, dysphonia, cough, malaise, fever, dental pain, halitosis or pain/discomfort in the ears); then polyposis is confirmed through endoscopic evaluation and imaging (10). Management of SNP requires adequate medical treatment that may be supplemented with surgery. Corticosteroids are the first-line treatment for chronic rhinosinusitis (CRS) with polyposis, according to the most recent European and North American consensus documents (11–13). The impact of surgical treatment is difficult to establish with precision, since surgery is performed on those patients who are intractable to medical management, while recent endoscopic surgery is associated with better results than conventional simple polypectomies (8,14). Approximately 10% of all patients undergoing endoscopic surgery show a poor response to surgical treatment and concomitant medical therapy (8). Furthermore, recurrence of polyps is a major concern, and can be classified according to the type of surgery, definition of recurrence, follow-up duration, disease extension, and background disorders (15-21). 

The purpose of this study was to assess the short-time outcome of endoscopic sinus surgery based on subjective clinical presentation of the four main symptoms of SNP including nasal obstruction, rhinorrhea, facial pain and olfactory disturbance over 2 years’ follow-up among patients with high-grade sinonasal polyposis.

## Materials and Methods

This prospective longitudinal cohort study included CRS patients followed up for at least 2 years after endoscopic sinus surgery. Between April 2008 and February 2011, 148 adult CRS patients with and without polyposis were followed from private practice and the institution clinic. Diagnosis of CRS with nasal polyps was based on the European Position Paper on Rhinosinusitis and Nasal Polyps (EPOS) 2012 criteria (11), as the indication for surgery. Patients with low-grade polyposis (Grade 1,2 according to the Lildholdt classification system), no chronic polypoid sinusitis, or who did not complete 2-year follow-up were excluded from the study. Sixty-two of the 148 original patients were finally included in the study. 

CT scan as the imaging technique was performed in all patients preoperatively and during follow-up, according to indication. The sensitivity and specificity of CT has been evaluated using the Lund-Mackay scoring system (22). Nasal endoscopic evaluation of SNP was performed according to the Lildholdt classification (23), and only those with advanced grade (Grade II or III) were selected. Patients were asked to complete the questionnaire relating to the four main symptoms of severity prior to surgery (preop) and 1,3,6,12 and 24 months after surgery (PO1, PO3, PO6, PO12, and PO24 m; respectively) (Table.1).

**Table 1:** Symptoms scoring system

	**Score** **1**	**Score** **2**	**Score** **3**	**Score** **4**	**Score** **5**
Nasal Obstruction	Absent	Occasionally Partial	Always Partial	Usually Complete	Always Complete
Facial Pain	Absent	Occasionally Vague	Always Vague	Occasionally Disturbing	Always Disturbing
Rhinorrhea	Absent	Occasionally Suffering	Usually suffering	Usually Disturbing	Always Disturbing
Olfactory Disturbance	Absent	Occasionally Hyposmia	Always Hyposmia Occasionally Anosmia	Usually Anosmia	Always Anosmia


Nasal endoscopic evaluation was performed during these periods of follow-up, and patients were also asked to undertake a CT scan if indicated. Subjects were given a thorough questionnaire, an assessment of the clinical management of the disease, and revision procedures as well as concomitant diseases and other conditions including allergic rhinitis, asthma, aspirin sensitivity, allergic fungal rhinosinusitis (AFRS), as well as active and passive smoking were recorded. All patients were evaluated for allergy by skin prick testing and for asthma by pulmonary function testing as performed by a pulmonologist. 

The four major symptoms of CRS (nasal obstruction, rhinorrhea, facial pain, and olfactory disturbance) were the primary outcome measures during post-surgery follow-up. All patients had a complete ethmoidectomy and maxillary antrostomy and additional procedures as indicated (Table.2).

**Table 2:** Surgical procedures distribution

**Surgical Type**	**Percent**
Anterior Ethmoidectomy	100
Posterior Ethmoidectomy	100
Antrostomy	100
Frontal Recess Management	91.93
Sphenoidotomy	77.42
Middle Turbinoplasty	29.03
Septoplasty	30.64
	

All surgery was performed under general anesthesia by the author. Any significant deviation of the nasal septum was corrected endoscopically at the same time or through a conventional approach. Antibiotics (azithromycin) and oral corticosteroids were prescribed for 7 days post-operatively along with saline douches. Following this, topical nasal steroids and saline douches as maintenance were recommenced for at least for 6 months and after that on case-by-case basis. The study was approved by the local Research Ethics Committee. The Statistical Package for Social Sciences (SPSS) version 13 was used for data analysis. Two-way repeated-measures analysis of variance (ANOVA) including the Mauchly test of sphericity, the Huynh-Feldt test and the multiple comparisons Bonferroni test were used to compare the effects of two factors when one of the factors is repeated-measures and the other is not.

Descriptive statistics (frequency, mean, and standard deviation) were determined for variables.

The mean scores in preop, and postoperative PO1, PO3, PO6, PO12, and PO24 months of each symptom were compared using the paired t-test. Only the p-value is presented in the results. Statistical significance was attributed when P<0.05.

## Results

Sixty-two patients with chronic polypoid rhinosinusitis who underwent endoscopic sinus surgery were enrolled in the study. 

Thirty-seven of the patients were men and 25 were women, with a mean age overall of 41.24±12.47 years (range, 13–65 years). All patients completed 2 years of follow-up. Patient information, comorbidities and presenting symptoms are described in Table. 3. 

**Table 3:** Patient information, comorbidity and presenting symptoms

		**Number**	**Percent (%)**
**Polyp Size**	Grade II	21	33.9
	Grade III	41	66.1
Allergic Rhinitis		34	54.8
Allergic Fungal Rhinosinusitis		6	9.67
Asthma		27	43.5
Samter Triad		4	6.5
Smoking		9	14.5
Passive Smokers		11	17.7
Major Symptoms	Obstruction	62	100.0
	Rhinorrhea	55	88.7
	Anosmia	28	45.2
	Hyposmia	27	43.5
	Facial Pressure	48	77.4
Early Complication	Major	1	1.6
	Minor	2	3.2
Reoperation		7	11.3


The severity of symptoms at the beginning of the study was classified according to polyposis grading (Table. 4).

**Table 4:** Symptoms scoring according to polyp grading

**Symptom**	**Grade**	**Number**	**Mean ± SD**	**P Value**
PND	2	21	3.00 ± 1.52	0.002
	3	41	4.00 ± 0.92	
Pressure	2	21	1.95 ± 1.16	0.005
	3	41	2.78 ± 0.99	
Smell Disturbance	2	21	2.48 ± 1.29	< 0.001
	3	41	4.41 ± 0.92	
Obstruction	2	21	3.05 ± 1.07	< 0.001
	3	41	4.39 ± 0.77	
				

**Fig 1:**
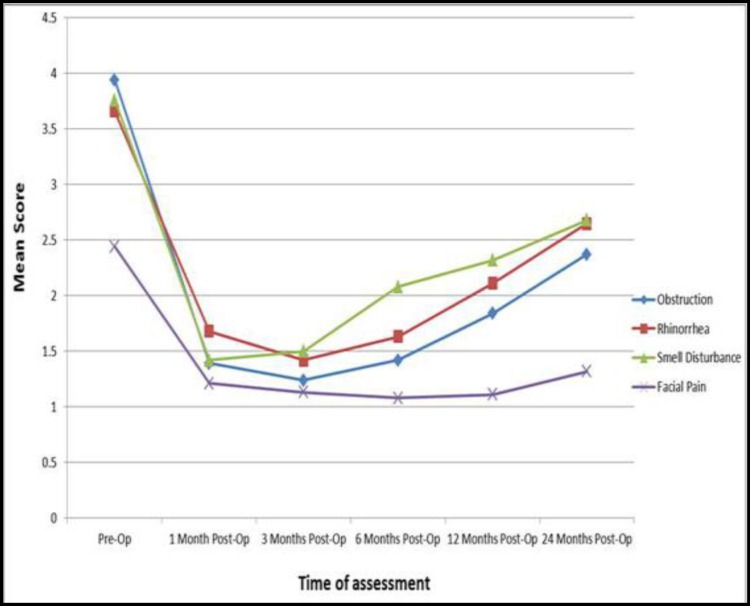
Symptoms severity changes during follow up

All major symptoms showed significant improvement after surgery (P=0.000), and symptom severity changed after operation according to the time of follow-up, as indicated by the Huynh-Feldt test (P=0.000). However these changes were not the same for all symptoms. Time and variance analysis showed that smell disturbance changed significantly earlier than other symptoms (P=0.008). Although the severities of major symptoms were diminished after surgery even at 24 months post-procedure; gradually certain symptoms did become more prominent post-surgery. The progression of disease differed according to symptom, such that initially sense of smell then rhinorrhea and finally obstruction as well as facial pain showed worsening, with a mean time of 6, 12 and 24 months after surgery, respectively (Fig.1).

Asthma, allergic rhinitis, AFRS and Samter’s triad were associated with worsening of the symptoms in a shorter-timeframe post-operatively (P<0.05). Smoking did not show such a correlation with symptom alteration during follow-up (P= 0.338) (Table. 5).

**Table 5:** Comorbidity and smoking effect on symptoms progression

	**Nasal Obstruction** **(P value)**	**Rhinorrhea** **(P value)**	**Smell Disturbance** **(P value)**	**Facial Pain** **(P value)**
Asthma	<0.001	<0.001	<0.001	<0.001
Allergic Rhinitis	0.026	<0.001	0.005	<0.001
Samter’s Triad	0.004	0.005	0.23	<0.001
AFRS	0.018	0.001	0.269	<0.001
Smoking	0.858	0.388	0.353	0.873
				

Polyp recurrence was noted in nine cases (14.51%). Minor recurrence (general edema of the mucosa or minor polyposis) was found in 32 patients (51.6%). The recurrence rate was related to associated diseases and the severity of symptoms at presentation (Table. 6).

**Table 6:** Recurrence according to grade and associated diseases

			**Polyp**	**Recurrence**	
		**No** **Polyp**	**Minor Polyposis**	**Frank Polyposis**	**P. Value**
Grade	II	12	8	1	0.016
	III	9	24	8	
Allergy	Yes	0	18	9	< 0.001
	No	21	14	0	
Asthma	Yes	1	17	9	<0.001
	No	20	15	0	
AFRS	Yes	0	2	4	0.001
	No	21	30	5	
Samter	Yes	0	0	4	<0.001
	No	21	32	5	
Smoking	Yes	1	8	0	0.051
	No	20	24	9	
PND	Mean±SD	3.10±1.48	3.88±1.04	4.22±0.83	0.042
Pressure	Mean±SD	1.76±0.96	2.66±0.79	3.67±1.23	<0.001
Smell Disturbance	Mean±SD	2.62±1.36	4.19±1.09	4.89±0.33	<0.001
Obstruction	Mean±SD	3.05±1.07	4.31±0.82	4.67±0.50	<0.001
					

Seven (11.3 %) of the 62 patients underwent revision surgery during follow-up. 

One case was due to frontoethmoidal mucocele formation, one was for middle-turbinate adhesion and blockage of the ostiomeatal complex (OMC) and the remaining cases had symptomatic frank polyposis. All cases except the mucocele case were among patients with AFRS or Samter’s triad.

Early surgical complications were seen among four patients as follows: one cerebrospinal fluid (CSF) rhinorrhea which was managed during surgery, two cases of periorbital ecchymosis and emphysema, and one case of nasal bleeding requiring a return to surgery.

## Discussion

Today, endoscopic sinus surgery is a widely accepted procedure for the management of sinonasal polyposis, and is a replacement for traditional, conventional nasal polypectomies. 

As we showed in our study, clinical presentation and quality of life improved significantly after surgery. As indicated in the literature and in this study, results on nasal symptoms show that SNP resistant to medical therapy is greatly improved by surgical treatment followed by treatment with a topical nasal steroid. However, nasal polyps treated either medically or surgically have a high recurrence rate, which is a major concern for each surgeon. Early detection, prevention and postponement of this condition are the aims for future works and studies in this domain. The rate of recurrence differs widely according to the extension of the primary disease, duration of follow-up, comorbidity, type of surgery, and definition of recurrence (8,15–21).

 In our study, nasal obstruction was seen among all patients; however, olfactory disturbances (either hyposmia or anosmia, rhinorrhea [anterior or posterior] and facial pain or pressure) were the most common symptoms among the majority of patients, in decreasing order of prevalence. The most frequent symptoms in patients with SNP have been shown to be nasal obstruction, smell alterations, rhinorrhea and facial pressure or pain, in this order (24). Nasal obstruction, rhinorrhea and facial pain are less specific symptoms for CRS, while olfactory disturbance and smell changes have been shown to be significantly more specific for CRS diagnosis (25). Furthermore, Litvak et al. showed the correlation between the degree of smell disturbance and the severity of CRS, as assessed by CT or nasal endoscopy (26). Another study on nasal mucosal biopsies has revealed a correlation between olfactory disturbance and degree of inflammation of the nasal mucosa (27).

Previous studies reported that the rates of smell improvement after endoscopic surgery in CRS varied from 23 to 85% (28–32). The current study found that 96.3% of all cases with SNP who had a problem with sense of smell preoperatively showed some improvement in the first 3 months after surgery, and only two cases with pre-operative anosmia showed no changes after the procedure; furthermore, seven cases had a normal olfactory function before surgery. In addition, we showed that changes in smell after endoscopic sinus surgery is a good and sensitive symptom and might show a progression to sinonasal edema; and therefore can be an early sign of recurrence. This is because we found the smell function tended to decline earlier than other symptoms, and thus could be a useful ‘early alarm symptom’ which may be reported by patients, notifying the surgeon of the need for early medical management to prevent disease progression to frank polyposis. Following a review of the medical literature, we were not able to find a comparable study with which to compare our results.

Comorbidities such as asthma, allergic rhinitis, AFRS and Samter’s triad were associated with more severe conditions as well as with a more rapid progression of symptoms post-operatively in our study.

Asthma was seen among 43.5% of our patients and of those, four patients showed aspirin sensitivity (Samter’s triad). These patients had more severe disease according to the symptoms score, and also rapid symptom progression after surgery compared with cases without these disorders. A literature review revealed that bronchial hyper-responsiveness and asthma are common (21–48%) in patients with SNP. This association increases in patients with NSAID intolerance (8). A Spanish study found asthma among 36% of patients with SNP, compared with 15.4% prevalence in subjects without this condition (24). The severity of the disorder is greater when SNP and asthma coexist (33). Furthermore, approximately 5–15% of all asthmatic patients may progress to polyposis (34,35). The prevalence of SNP among Samter’s triad can reach 70% (34). Comorbid Samter’s triad is a particularly serious condition, due to its inadequate response to treatment and its high recurrence rate (36). In addition, patients with asthma associated with SNP tend to have a poorer perception of control of the disease, due to the persistence and severity of the associated sinonasal symptoms(8). Therefore, management of the upper airway disease must not be ignored.

Allergic sensitization in patients with SNP varies between 10–96.5% (8). Also 0.5–4.5% of all patients with allergic rhinitis, have SNP (34,37); similar to the rate seen among the general population (38). Like our study, some other studies have reported the prevalence of atopy to be greater among SNP patients; however other authors have observed no such association (34,37). Nevertheless, in patients with both conditions, the management of allergy has been shown to have a positive impact on the symptoms of SNP (33). In total, 54.8% percent of our patients showed a positive response to at least one allergen during a skin prick test. The rate of allergic rhinitis according to medical history and a positive prick test among the normal population and CRS (including polyposis and none polyposis type) in our region was reported at 22.5% and 64% (39,40), respectively. This finding reinforces the observation that allergy may be a predisposing factor for SNP. Studies in Spain have reported a prevalence of 63% and 48% (41,42). Therefore, it is advisable to investigate the presence of allergic sensitization in patients with SNP, based on skin tests or specific IgEs, according to international standards. The treatment of coexisting allergic rhinitis improves the symptoms of patients with SNP (42,43).

Allergic fungal sinusitis (AFS) is a noninvasive form of fungal rhinosinusitis with a prevalence of 6–9% among rhinosinusitis cases requiring surgery. The fungi responsible for AFRS show great diversity and regional variation, and the incidence of AFS has been reported worldwide (44). The prevalence of AFRS in our region was reported to be 9.45% among patients with SNP (45). The rate of recurrence after surgery was reported to be more common than cases without this condition among different studies. In our survey, 8.7% of SNP patients fulfilling the AFRS criteria had more severe disease according to CT scan findings and clinical presentation. Furthermore, 50% of these patients underwent revision surgery during 2 years of follow-up. This was significantly more common than in cases without this condition.

In general, a review of the literature suggests that smoking is less common among patients with SNP than in the general population. Rugina (46) reported a smoking prevalence of 15.5% in patients with SNP, versus 35% in the general population among the French population. Likewise, in a Spanish population, Toledano et al. (47) observed a 25.5% prevalence of smokers among patients with SNP, versus 38.9% in healthy individuals. In our patient group, 14.5% and 17.7% of patients were smokers and passive smokers, respectively. The rate of smoking in our general population was not reported for the purposes of comparison. This factor did not influence postoperative recovery and recurrence rates. This may be due to cessation or at least decrease in smoking habits after surgery through the surgeon’s recommendation to the patients.

Polyp size and extension have been shown to be an important prognostic factor, as well as recurrence rate of the disease (48); evaluation of this aspect therefore appears important in establishing the clinical diagnosis of SNP. Several SNP size and extent grading scales have been proposed and evaluated for reproducibility and inter-individual variability (49). The grading system proposed by Lildholdt has been recommended as one of the best methods for evaluating the evolution of the size of the nasal polyps (23), and was used by the author. The grades of our patients were high according to this classification, and all were selected from Grade II and III because we had planned to compare symptom progression during the study. One of the reasons for some differences between our study and others could be selection bias. However, in patients subjected to surgery due to SNP, nasal endoscopy was not seen to correlate well to the symptoms scores (50).

Today, endoscopic sinus surgery will continue to play an important role in the management of SNP in cases who are intractable, in order to achieve medical management with improvement of symptoms and quality of life. Nevertheless, although meticulous surgery may reduce the percentage of recurrences, there are cases where even the most careful complete removal of the entire pathology cannot prevent recurrence. Recurrence may be linked to intrinsic unknown factors, and some of the negative prognostic factors which might be implicated in recurrence may be allergy and asthma, as well as fungal and aspirin sensitivity. While this present study extrapolated these factors to some extent, there is much research still required to recognize the exact related intrinsic and extrinsic factors.

## Conclusion

Endoscopic sinus surgery significantly improved the major symptoms and quality of life of patients with sinonasal polyposis. Patients had acceptable levels of medical management of their condition and few required re-operation within 2 years of surgery. Decline in smell function could be a good indicator for early sinonasal edema before formation of frank polyposis. Certain comorbidities such as asthma, allergic rhinitis, as well as sensitivity to fungus and aspirin, should be considered as factors for early recurrence. Finally, these same factors warrant close observation and concern for patients undergoing surgery.
